# miR-16-5p Is a Stably-Expressed Housekeeping MicroRNA in Breast Cancer Tissues from Primary Tumors and from Metastatic Sites

**DOI:** 10.3390/ijms17020156

**Published:** 2016-01-26

**Authors:** Gabriel Rinnerthaler, Hubert Hackl, Simon Peter Gampenrieder, Frank Hamacher, Clemens Hufnagl, Cornelia Hauser-Kronberger, Franz Zehentmayr, Gerd Fastner, Felix Sedlmayer, Brigitte Mlineritsch, Richard Greil

**Affiliations:** 1 IIIrd Medical Department with Hematology and Medical Oncology, Oncologic Center, Paracelsus Medical University Salzburg, Salzburg 5020, Austria; g.rinnerthaler@salk.at (G.R.); s.gampenrieder@salk.at (S.P.G.); Frank.Hamacher71@gmx.de (F.H.); cl.hufnagl@salk.at (C.H.); b.mlineritsch@salk.at (B.M.); 2 Salzburg Cancer Research Institute with Laboratory of Immunological and Molecular Cancer Research and Center for Clinical Cancer and Immunology Trials, Salzburg 5020, Austria; 3 Cancer Cluster Salzburg, Salzburg 5020, Austria; 4 Division of Bioinformatics, Biocenter, Medical University of Innsbruck, Innsbruck 6020, Austria; hubert.hackl@i-med.ac.at; 5 Department of Pathology, Paracelsus Medical University Salzburg, Salzburg 5020, Austria; c.kronberger@salk.at; 6 Department of Radiotherapy, Paracelsus Medical University Salzburg, Salzburg 5020, Austria; f.zehentmayr@salk.at (F.Z.); g.fastner@salk.at (G.F.); f.sedlmayer@salk.at (F.S.)

**Keywords:** miR-16, miR-16-5p, microRNA, endogenous control, housekeeper, control, breast cancer

## Abstract

For quantitative microRNA analyses in formalin-fixed paraffin-embedded (FFPE) tissue, expression levels have to be normalized to endogenous controls. To investigate the most stably-expressed microRNAs in breast cancer and its surrounding tissue, we used tumor samples from primary tumors and from metastatic sites. MiRNA profiling using TaqMan^®^ Array Human MicroRNA Cards, enabling quantification of 754 unique human miRNAs, was performed in FFPE specimens from 58 patients with metastatic breast cancer. Forty-two (72%) samples were collected from primary tumors and 16 (28%) from metastases. In a cross-platform analysis of a validation cohort of 32 FFPE samples from patients with early breast cancer genome-wide microRNA expression analysis using SurePrintG3 miRNA (8 × 60 K)^®^ microarrays from Agilent^®^ was performed. Eleven microRNAs could be detected in all samples analyzed. Based on NormFinder and geNorm stability values and the high correlation (*rho* ≥ 0.8) with the median of all measured microRNAs, miR-16-5p, miR-29a-3p, miR-126-3p, and miR-222-3p are suitable single gene housekeeper candidates. In the cross-platform validation, 29 human microRNAs were strongly expressed (mean log2-intensity > 10) and 21 of these microRNAs including miR-16-5p and miR-29a-3p were also stably expressed (CV < 5%). Thus, miR-16-5p and miR-29a-3p are both strong housekeeper candidates. Their Normfinder stability values calculated across the primary tumor and metastases subgroup indicate that miR-29a-3p can be considered as the strongest housekeeper in a cohort with mainly samples from primary tumors, whereas miR-16-5p might perform better in a metastatic sample enriched cohort.

## 1. Introduction

MicroRNAs are small, approximately 22 nucleotides long non-coding single-stranded RNAs, regulating gene expression at a post-transcriptional level. The human genome may encode more than 1000 microRNAs and approximately 60% of human genes are regulated by microRNAs thereby controlling cell proliferation, apoptosis, differentiation and angiogenesis [1]. Consequently, microRNAs can play a distinct role in tumorigenesis and altered microRNA expression profiles were described in different malignancies [2]. MicroRNA genes are frequently (more than 50%) located in cancer-associated fragile regions and break points of the DNA [3]. Generally, microRNAs act as tumor suppressors, which negatively regulate oncogenes, genes that promote cell proliferation, as well as genes that inhibit cell division [4,5].

MicroRNA expression analysis can be performed by semi-quantitative methods like Northern blotting [6], bead-based flow-cytometry [7] and hybridization with locked nucleic acid probes (arrays). Hybridization platforms are commercially available from Affimetrix^®^ and Agilent^®^ (SurePrint Human miRNA Microarray platform) [8,9].

For a quantitative reproducible microRNA expression profiling, real-time quantitative PCR (qPCR) has become the method of choice. High-throughput microRNA profiling qPCR plattforms from several companies (Exiqon, Life Technology—TaqMan^®^ microRNA array, Quiagen, Quanta BioSciences, and WaferGen) are available. Next generation sequencing, in particular small RNA-seq, can also be performed for quantitative microRNA expression profiling, but these methods are mainly used for discovery applications. In addition, a hybridization technique with tagged probes in solution (nCounter from Nanostring^®^) can be used for quantitative microRNA analysis [9,10,11].

A cross-platform comparison of microRNA expression results should be interpreted with caution, because there is a discordance of expression levels between different available qPCR, hybridization, and sequencing platforms. In a quality control study comparing expression results of 12 available commercial platforms, the average concordance between any two platforms was 86.7% (95% CI, 86.0%–87.3%). When the detection rate was taken into account, the concordance dropped to 79.2% (95% CI, 77.0%–80.4%). Furthermore, accuracy, reproducibility, specificity and sensitivity varied between different platforms. The authors concluded that each application has its strengths and weaknesses, and the selection of a microRNA platform should depend on study goals [12].

For quantification, raw expression levels have to be normalized to reduce false positive or negative data values due to variations in pre-analytic and analytic procedures, and especially due to biological variations [11].

Several microRNA expression data normalization strategies have been postulated [11]:

(1) Raw expression levels can be normalized to endogenous controls like housekeeping genes (microRNAs). These are expected to show small variation and high correlation to the mean (median) of all measured microRNAs, because the majority of microRNAs are not changing and mean normalization might be appropriate. The difference (Δ*C*q) between the PCR-derived cycle threshold (*C*q) of the target microRNA and the *C*q value of the endogenous control is used for relative microRNA quantification [13]. There is currently no consent on suitable endogenous controls for microRNA profiling from FFPE tissue. For Taqman^®^ human microRNA cards, the small nuclear RNA (snRNA) U6 and the small nucleolar RNAs (snoRNAs) RNU44 and RNU48 are recommended as endogenous controls based on healthy tissue and tumor cell line studies (NCI-60). However, these small RNAs have different biological und biochemical characters [14] compared to microRNAs and extraction quality, reverse transcription and PCR amplification may differ also from that of microRNAs [14,15]. Since normalization to small RNAs could therefore introduce bias, endogenous controls belonging to the same class of RNAs are likely more suitable housekeepers. In a comprehensive study by Davoren *et al.*, eight small RNAs previously described as endogenous controls for microRNA analysis in malignant tissue were analyzed in malignant, benign and healthy breast tissue [16]. Out of three snoRNAs (RNU19, RNU48 and Z30) and five microRNAs (let-7a-5p, miR-10b-5p, miR-16-5p, miR-21-5p, and miR-26b-5p), let-7a-5p and miR-16-5p were identified as the most stably expressed RNA pair.
(2) A further normalization strategy is to normalize to an exogenous spike-in reference gene, which can be introduced at different analysis steps. The *C. elegans* microRNA cel-miR-39 [17,18] is the most frequently used non-human microRNA for this purpose. This method adjusts deviation in the environmental process but does not correct for variances in sampling and sample quality. Therefore, normalization to a spike-in control has its strengths in quality control and calibration, but is limited in comparative microRNA expression analysis [11].
(3) Absolute normalization of expression levels by calculating absolute concentrations on the basis of calibration curves does not consider the influence of RNA quality. Therefore, this method is not optimal for quantification of microRNA and is only feasible for samples with a good RNA quality [11].

As microRNA expression analyses of FFPE cancer tissue without microdissection are influenced by the microenvironment, and surrounding tissue normalization to endogenous controls can be considered as standard procedure in cancer research. In this study, we investigated the most stably-expressed microRNA in breast cancer tissues from primary and metastatic sites.

## 2. Results

Eleven microRNAs (Table 1) could be detected in all 58 samples from primary tumor and metastatic sites. Four of these microRNAs (miR-16-5p, miR-29a-3p miR-126-3p, miR-222-3p) showed also a high correlation with the median of all measured microRNAs (Spearman rank correlation *rho* ≥ 0.8) (Table S1, Supplementary Material).

**Figure 1:**
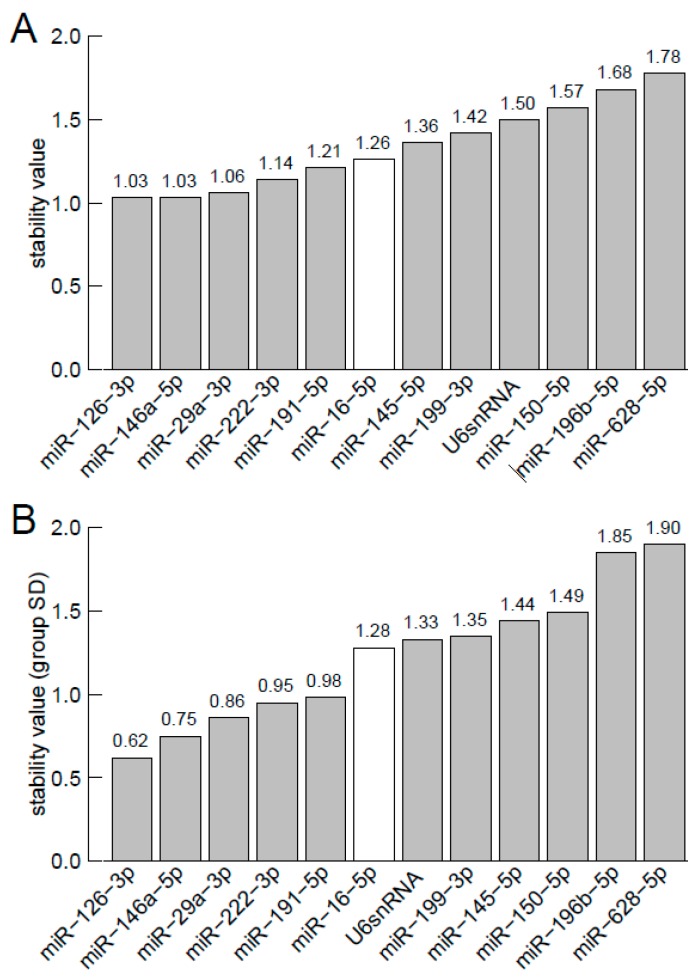
miRNA/ncRNA expression stability by geNorm (**A**) and NormFinder (**B**). (**A**) Average expression stability value *M*, excluding the given microRNA in a stepwise procedure, thereby ranking from the least stable control candidate right to most stable control right; (**B**) Stability values for the 12 control candidates gives an estimation of the intragroup variance (only one combined group is considered) ranked from the most stable (left) to the least stable candidate (right).

**Figure 2:**
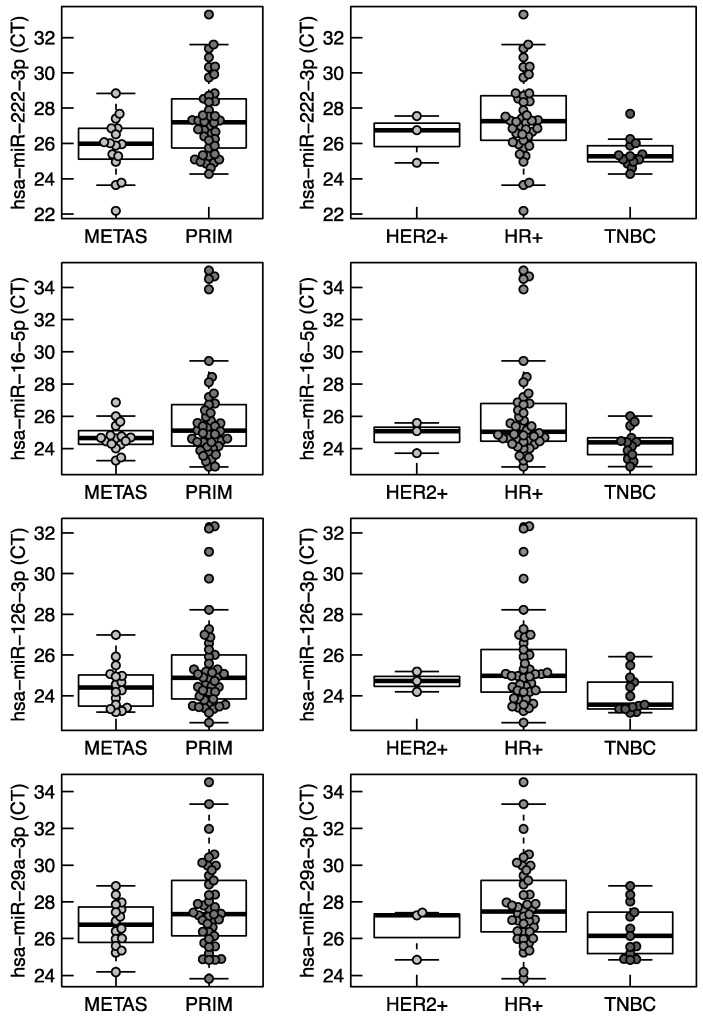
Boxplots of miRNA expression of selected housekeeper candidates per subgroup. METS: samples derived from metastasis; PRIM: samples derived from primary; HER2+: HER2 positive; HR+: hormone receptor positive; TNBC: triple negative breast cancer.

The small nuclear RNA U6 (snU6), an endogenous control on TaqMan^®^ Array Human MicroRNA Cards, was consistently expressed across all samples (coefficient of variation CV = 11.7%). The identified microRNAs showed even more consistent expression levels with a CV from 5.5% to 10.8%. Gene stability values according to geNorm analysis [19,20] and NormFinder [21], with lower values indicating increased gene stability across samples, were also lower for most of the eleven microRNA housekeeper candidates compared to snU6 (Figure 1A, Figure 1B, Table 1, Tables S2 and S3 (Supplementary Material)). NormFinder stability values calculated across the primary tumor and metastasis subgroup, showed highest gene stability for miR-16-5p in the metastasis subgroup and for miR-126-3p in the primary tumor subgroup. As illustrated by boxplots (Figure 2 for miR-16-5p, miR-29a-3p miR-126-3p, miR-222-3p and Figure S1 (Supplementary Material) for all other housekeeper candidates), median *C*_t_-values of miR-16-5p were most consistent between different subgroups (*i.e.*, primary tumor, metastasis, hormone receptor positive, HER2 positive, triple negative). Out of the 12 candidates miR-222-3p (*p* = 0.008) and miR-146a-5p (*p* = 0.006) showed even significant different expression between the breast cancer subtypes (Table S4, Supplementary Material).

Using geNorm, the most reliable combination of different microRNAs as endogenous controls was determined by a stepwise procedure. Where microRNAs are sequentially included into the normalization factor according to their increasing stability value (*M*-value), a pairwise variation between normalization factors consisting of a different number of housekeeper candidates was calculated. A combination of six microRNAs (miR-126-3p, miR-146a-5p, miR-29a-3p, miR-222-3p, miR-191-5p and miR-16-5p) seems to be most reliable for normalization according to this analysis. When using a combination of two microRNAs, miR-126-3p and miR-146a-5p showing an average expression stability M of 1.02 performed best. Detailed data of this geNorm analysis are provided in Table S3 and Figure S2 (Supplementary Material).

Based on NormFinder and geNorm stability values and the high correlation (*rho* ≥ 0.8) with the median of all measured microRNAs, miR-16-5p, miR-29a-3p miR-126-3p and miR-222-3p are suitable single gene housekeeper candidates.

**Table 1:** MicroRNA expressions of housekeeper candidates.

MicroRNA	Mean	Median	SD	CV (%)	*Rho*	BRCA *	geNorm	NormFinder **		
						CV (%)	M	All	Prim	Metas
hsa-miR-222-3p	26.97	26.77	2.13	7.88	0.86	21.6	1.14	0.95	0.63	1.29
hsa-miR-16-5p	25.70	24.84	2.78	10.80	0.83	9.3	1.26	1.28	1.42	0.61
hsa-miR-126-3p	25.17	24.70	2.08	8.26	0.80	9.6	1.03	0.62	0.50	0.86
hsa-miR-29a-3p	27.46	27.23	2.12	7.73	0.80	6.0	1.06	0.86	0.80	1.02
hsa-miR-146a-5p	27.82	27.54	2.00	7.18	0.76	20.3	1.03	0.75	0.67	0.93
hsa-miR-191-5p	25.21	24.68	2.42	9.60	0.71	10.8	1.21	0.98	1.06	0.74
U6snRNA	19.14	18.76	2.24	11.73	0.69	**–**	1.50	1.33	1.48	0.87
hsa-miR-199-3p	28.68	28.50	2.33	8.13	0.68	8.9	1.42	1.35	1.31	1.48
hsa-miR-628-5p	33.16	33.17	1.83	5.52	0.64	34.4	1.78	1.90	1.98	1.51
hsa-miR-145-5p	27.59	27.18	2.47	8.95	0.60	10.4	1.36	1.44	1.48	1.35
hsa-miR-150-5p	27.94	27.80	2.23	7.98	0.58	20.4	1.57	1.49	1.44	1.62
hsa-miR-196b-5p	31.63	31.62	2.03	6.43	0.50	20.4	1.68	1.85	1.49	1.84

SD: standard deviation; CV: coefficient of variation; *rho*: Spearman rank correlation, microRNAs with a *rho* ≥ 0.8 are greyed out; M: average expression stability value; PRIM: primary tumor; METAS: Metastasis; ALL: all 58 patients; * microRNAseq data of the The Cancer Genome Atlas (TCGA) breast adenocarcinoma (BRCA) analysis (including 755 patients); ** Stability values from NormFinder analysis are given based on the estimated intragroup variance for the given group.

### Cross-Platform Validation

Based on a mean log2-intensity >10 (corresponding to a signal intensity of >1024), 29 human microRNAs could be filtered. Twenty-one of these microRNAs showed a coefficient of variation <5%. Ten of these miRNAs can be also found on TaqMan Human MicroRNA array A and B Cards Set v3.0 (Table 2), and two of them (miR-16-5p and miR-29a-3p) were also selected as well-suited endogenous candidates as described above (Table 1).

**Table 2:** MicroRNA expressions of validation cohort.

MicroRNA *	Mean	SD	CV (%)
hsa-let-7f	11.55	0.30	2.56
hsa-miR-638	11.68	0.38	3.23
hsa-let-7g	10.49	0.35	3.38
hsa-let-7i	10.34	0.37	3.62
hsa-miR-26a	10.09	0.37	3.67
hsa-let-7a	12.43	0.46	3.70
hsa-miR-16-5p	11.01	0.41	3.74
hsa-miR-494	13.88	0.53	3.85
hsa-miR-23a-3p	10.43	0.44	4.22
hsa-miR-29a-3p	10.23	0.44	4.34

SD: standard deviation; CV: coefficient of variation, microRNAs with a high correlation with the median of all measured microRNAs (*rho* ≥ 0.8) in the main cohort are greyed out; * present also on TaqMan Human MicroRNA array A and B Cards Set v3.0.

## 3. Discussion

Due to their diagnostic, prognostic and predictive potential in cancer research, there is an increasing amount of published microRNA studies. Besides expression analysis from tumor tissue (FFPE or fresh frozen samples), biomarker studies of circulating microRNAs in the blood are frequently conducted. Since the stability of microRNAs is insusceptible to changes of pH, temperature and mechanical influences as well as resistant to RNase and freeze-thaw cycles, their detection in body fluids is feasible [22].

In cancer research, microRNA analyses are in general performed with RNA derived from a cellular mixture and not from micro-dissected or sorted cells. Tumor tissue and, in particular, metastatic lesions of solid tumors, represent a composition of different cells: cancer cells, cells belonging to the so-called tumor microenvironment (blood vessels, immune cells, fibroblasts, *etc.*) and cells from adjacent healthy tissue [23]. Furthermore, the cellular content and the genetic profile varies between different metastatic sites and the primary tumor but also between different sections of a solid tumor or metastatic lesion [24]. Because of this inter- and intra-tumor heterogeneity, expression normalization for comparative microRNA analysis is crucial, not only for inter-patient, but also for intra-patient comparisons.

In breast cancer patients, metastatic disease is not always histologically confirmed. This is often the case in patients with synchronous metastases where, outside of clinical trials, such sampling at multiple sites would not seem justified as well as in patients with metachronous disease, if biopsies would appear dangerous, without chance of adequate yield, or unnecessary due to clinical reasons. Hence, for breast cancer, simultaneously collected paired samples from primary and different metastatic sites are rarely available. To our knowledge, the present study is the first analysis of the most stably-expressed microRNA in breast cancer tissues from primary and metastatic sites.

In our study, 11 out of 754 microRNAs were detected in all 58 samples analyzed. Expression levels of four of these microRNAs (miR-16-5p, miR-29a-3p, miR-126-3p, and miR-222-3p) showed also a high correlation with the median of all measured microRNAs (*rho* ≥ 0.8) and, therefore, might be well suited as endogenous controls. These microRNAs also showed low stability values, as determined by geNorm (1.03–1.26) and NormFinder (0.62–1.28), two commonly used tools for analysis of housekeeper candidate stability.

Despite the stable expression of miR-16-5p, miR-29a-3p miR-126-3p, and miR-222-3p in our patient cohort, these microRNAs have distinct functions in breast cancer. In a microRNA expression profiling of 20 different breast cancer samples, representing common breast cancer phenotypes, an association with HER2, estrogen (ER) and progesterone receptor (PR) status was shown [25]. miR-126-3p expression was associated with HER2 status and miR-222-3p expression with PR status. In contrast, miR-16-5p and miR-29a-3p expressions were independent of HER2, ER, and PR status. Additionally, miR-126-3p was differentially expressed between luminal A and luminal B intrinsic subtypes in a microRNA expression analysis of 93 primary human breast tumors [26]. In a case study of 456 triple negative breast cancer (TNBC) patients, high levels of miR-126p-3b were independently associated with favorable outcomes [27]. In another study of 173 TNBC patients, a microRNA signature including miR-16-5p was associated with prognosis [28]. An up-regulation of miR-126-3p was associated with a favorable outcome in ER positive tumors of 87 breast cancer patients [29]. Especially altered expression of miR-29a-3p, miR-126-3p, and miR-222-3p, but also of miR-16-5p can be involved in breast cancer development, tumor spread, proliferation and drug resistance (Table 3). Furthermore, miR-16-5p has been identified as regulator of osteolytic bone metastasis [30]. In our dataset, miR-16-5p was the most consistent expressed housekeeper candidate between different subtypes (*i.e.*, hormone receptor positive, HER2 positive, triple negative) as illustrated by a boxplot (Figure 2).

**Table 3:** Targets and functions in breast cancer of housekeeper candidates.

MicroRNA	Variation *	Targets	Function	References
miR-16-5p	down-regulated	FEAT (faint expression in normal tissues, aberrant overexpression in tumors)	Tumor, suppressor	[31,32,33]
		CCND1 (Cyclin D1)		
		BCL2 (B-cell lymphoma 2)		
		RPS6KB1 (Ribosomal protein S6)		
miR-29a-3p	up-regulated	TTP (tristetraprolin)	MetastamiR, OncomiR (drug resistance)	[34,35]
		PTEN		
miR-126-3p	down-regulated	VEGF	Tumor, suppressor, MetastamiR	[36,37,38,39,40,41]
		PIK3R2 (phosphoinositide-3-kinase regulatory subunit 2)		
		IRS-1 (Insulin receptor substrate 1)		
		adapter molecule Crk		
		SDF-1α (stromal cell-derived factor-1 alpha)		
		KRAS		
miR-222-3p	up-regulated	ERα	OncomiR (drug resistance)	[35,42,43]
		p27^Kip1^ (cyclin-dependent kinase inhibitor 1B)		
		p57 (cyclin-dependent kinase inhibitor 1C)		
		TIMP3 (tissue inhibitor of metalloproteinase-3)		

* As compared with normal tissue or parental cell lines in case of preclinical data.

miR-16 has been previously described as a stable endogenous control for microRNA expression analysis from breast cancer tissue [16], but also from blood samples [44,45]. In 21 malignant, five benign and five normal breast tissue samples, an expression analysis of five microRNAs (let-7a, miR-10b, miR-16, miR-21 and miR-26b) and three snoRNAs (RNU19, RNU48 and Z30) was performed. Let-7a and miR-16-5p were most stably expressed with stability values of 0.312 and 0.379 using NormFinder and 1.327 and 1.473 using geNorm, respectively. The combination of let-7a and miR-16-5p achieved lowest stability values of 0.221 using NormFinder and 0.978 using geNorm [16].

miR-16-5p and miR-29a-3p are both strong housekeeper candidates. Taking consistency of median expression between different breast cancer subgroups, the low stability value (NormFinder) of the metastasis subgroup, as well as the stable expression of miR-16-5p on TaqMan^®^ Array Human MicroRNA Cards and on SurePrintG3 Human miRNA microarrays from Agilent*^®^* in our analysis*,* and a stable expression in the TCGA dataset into account, miR-16-5p seems to be the most suitable endogenous control for microRNA expression in a metastatic sample enriched cohort. In a cohort of mainly samples from primary tumors, miR-29a-3p can be considered as the strongest housekeeper due to the low stability value (NormFinder) in the primary tumor subgroup.

## 4. Experimental Section

### 4.1. Patients and Study Design

Patients with metastatic breast cancer treated at our institution between 2006 and 2012 with first-line chemotherapy were identified for a predictive biomarker analysis for a bevacizumab response [46]. All 58 patients of the mentioned ongoing biomarker study, in whom a genome-wide microRNA profiling was performed, were included in this housekeeper analysis. Patient characteristics are shown in Table 4.

In another ongoing study, a genome-wide microRNA expression analysis using SurePrintG3 Human miRNA (8 × 60 K)^®^ microarrays from Agilent^®^ was performed in 32 patients with early breast cancer who had a radiotherapy after breast conserving surgery at the Department of Radiotherapy of the Paracelsus Medical University Salzburg. Expression data of those samples serve as a cross-platform validation for the present housekeeper analysis.

**Table 4:** Patient characteristics.

Characteristic		N	%
**Histology**	Ductal	43	74.1%
	Lobular	11	19.0%
	Others and unknown	4	6.9%
**Grade**	1	1	1.7%
	2	34	58.6%
	3	22	37.9%
	Unknown	1	1.7%
**Receptor status**	Hormone receptor positive	44	75.9%
	HER2 positive	3	5.2%
	Triple negative	13	22.4%
**Sample type**	Primary tumor	42	72.4%
	Metastasis	16	27.6%
	Biopsy	22	37.9%
	Resection	36	62.1%

### 4.2. Tissue Samples

Formalin-fixed paraffin-embedded (FFPE) tissue blocks containing samples from primary tumors, or if available, from metastatic sites, were selected by an experienced breast pathologist (C.K). Forty-two (72%) samples came from primary tumor and 16 (28%) from metastasis (three lymph node metastases, three liver metastases, two lung metastases, one pleural metastasis, two soft tissue metastases, three skin metastases, one ovarial metastasis, and one bone marrow infiltration). Twenty-two (38%) specimen were achieved by core biopsy and 36 (62%) by surgery. All tissue samples were collected prior to the start of first-line chemotherapy for metastatic disease. Three to five 10-µm sections were cut from each block without micro- or macro-dissection and placed in sterile Eppendorf tubes.

In the validation cohort, FFPE samples from primary tumors were selected by an experienced breast pathologist (CH). All samples were achieved by surgery and processed according to routine procedures immediately after surgery. Seven consecutive sections with a slice thickness of 2–4 µm were cut from each block without micro- or macro-dissection and placed in sterile Eppendorf tubes.

### 4.3. MiRNA Expression Analysis

TaqMan^®^ Array Human MicroRNA Cards (Applied Biosystems™, Waltham, MA, USA): Total RNA was purified from FFPE-Tissue using the *mir*Vana™ (Ambion™, Waltham, MA, USA) miRNA Isolation Kit and 1 µg was reverse transcribed to cDNA using the TaqMan*^®^* Reverse Transcriptase Kit (Applied Biosystems™, Waltham, MA, USA) according to the manufacturer’s instructions. TaqMan^®^ Human MicroRNA array A and B Cards Set v3.0 (Applied Biosystems™, Waltham, MA, USA) was used to quantify the expression of 754 human miRNAs.

SurePrintG3 Human miRNA microarrays from Agilent Technologies (Santa Clara, CA, USA): By means of micro-array technology, a panel of 1250 microRNA was screened. Isolation of total microRNA and chip-based micro-arrays (Agilent’s Sure PrintG3 Human miRNA microarrays) were performed according to standard procedures by the Comprehensive Biomarker Center™, Heidelberg, Germany.

### 4.4. Statistical Analysis

#### 4.4.1. TaqMan^®^ Array Human MicroRNA Cards

For the 754 human microRNAs expression (*C*_t_ values) were averaged over two replicates, microRNA expression in samples with *C*_t_ ≥ 40 were considered as not detected (and interpreted as missing value). Only microRNAs were considered as endogenous control if they could be detected in all samples from the 58 patients.

#### 4.4.2. MiRNAseq Analysis from the Cancer Genome Atlas (TCGA)

For TCGA breast cancer analysis, BRCA level3 miRNAseq (Illumina HiSeq) data were downloaded using Firehose/Firebrowse [47]. For TCGA, pan-cancer analysis microRNAseq (Illumina Hiseq), data were downloaded via the Synapse project (syn1695378) [48]. Only primary tumors were considered and normalized RPM values (reads per million miRNA mapped) were log transformed (log_2_(RPM+1)). Variability (*i.e.*, coefficient of variation) for >1000 precursor (stem-loop) microRNAs across all patient samples were calculated.

#### 4.4.3. SurePrintG3 Human miRNA Microarrays from Agilent^®^

Agilent microRNA array data were pre-processed and filtered using the AgiMicroRna Bioconductor library (as described in [49]). MicroRNAs showing a signal > (MeanNeg + 1.5 × SDNeg) in 100% of the samples were further considered and filtered for highly expressed microRNAs (mean log2-intesities > 10).

All statistical analyses and calculations were done using the R statistical software environment [50]. Several measures—including mean, median, standard deviation (SD), coefficient of variation (CV), and Spearman rank correlation coefficient (*rho*) against mean (median)—were calculated for all microRNAs (ncRNAs), which could be detected in all samples. MicroRNA expression (*C*_t_-values) were compared between primary tumors and metastasis using boxplots and Wilcoxon rank-sum test as well as between triple-negative breast cancer (TNBC), hormone receptor positive (HR+), and HER2 positive breast cancer (HER2+) using boxplots and Kruskal–Wallis tests. In addition, *p*-values were adjusted for multiple hypothesis testing based on the false discovery rate using the Benjamini–Hochberg method.

To further characterize stable expressed microRNAs (ncRNAs), an implementation in R of two commonly applied algorithms, namely NormFinder [19,51] and GeNorm [17,18,52] were used. Relative quantities were calculated in relation to the overall minimal *C*_t_ values (RQ = 2^min*C*t − sample*C*t^). In geNorm, the expression stability measure is calculated based on pairwise comparison between endogenous controls. For combining control candidates, the average expression stability measure *M* was calculated by stepwise removing the least stable control. Optimal number of control candidates was calculated based on pairwise variations between normalization factors (*V*). In a stepwise procedure, microRNAs according to their increasing stability value are subsequently included in the normalization factor. It is considered that there is no practical need to include the control candidate if *V* drops below 0.2. The stability value across the two groups, primary tumors and metastatic sites were evaluated using NormFinder.

### 4.5. Ethics

The study was approved by the Ethics Committee of the Province Salzburg (IRB number: 415-EP/73/67-2011 and 415-EP/73/85-2012).

## 5. Conclusions

In breast cancer, miR-16-5p is stably expressed both in samples from primary tumors and from metastatic sites and might be considered as the most relevant housekeeping microRNA. Therefore, miR-16-5p can be recommended as an endogenous control for normalization in microRNA expression analyses using breast cancer tissue.

## Supplementary Materials

Supplementary materials can be found at http://www.mdpi.com/1422-0067/17/2/156/s1.
